# Development of a Predictive Classification Model for Surfactant-Induced Skin Irritation

**DOI:** 10.1021/acsomega.5c07338

**Published:** 2025-11-13

**Authors:** Manuela Lechuga, Pedro A. García, Ana I. García-López, Cristina Tapia-Navarro, Francisco Ríos

**Affiliations:** † Department of Chemical Engineering, Faculty of Sciences, 16741 University of Granada, Campus Fuente Nueva S/N, Granada 18071, Spain; ‡ Department of Statistics and Operations Research, Faculty of Sciences, University of Granada, Campus Fuente Nueva S/N, Granada 18071, Spain

## Abstract

This study investigates the chemical properties of surfactants that significantly influence skin irritability using a predictive classification approach based on multiple linear regression and conditional inference trees. A data set comprising irritation values (Zein number, ZN) for 20 commercial surfactants and their binary mixtures was generated using an in vitro zein test. Key variables (hydrophilic–lipophilic balance (HLB), surfactant concentration, and ionic character) were evaluated to build robust statistical models. The multiple regression model explained 80% of the variability in skin irritation (adjusted *R* ^2^ = 0.801), while the classification tree achieved an overall accuracy of 72%, with precision and recall values of 0.70 and 0.68, respectively. The results highlight the hierarchical influence of surfactant properties, with HLB emerging as the most significant predictor, followed by concentration and ionic character. Notably, mixtures of anionic and nonionic surfactants showed reduced irritation potential compared to individual anionic surfactants. These findings offer valuable insights for the formulation of safer and more effective surfactant-based products.

## Graphical abstract

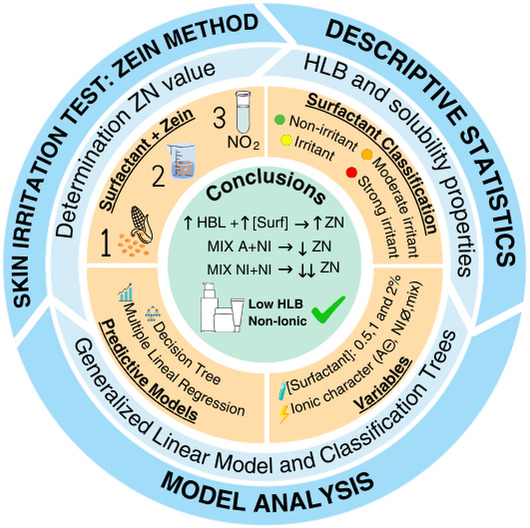

## 1 Introduction

Surfactants are molecules that have a lipid-soluble segment (soluble in fats) and another water-soluble segment (soluble in water or polar solvents), which makes them partially soluble in both water and fats, allowing them to occupy the interfaces. The properties and uses of surfactants come from two fundamental characteristics: on the one hand, their ability to adsorb at interfaces and, on the other hand, their tendency to associate to form organized structures. These properties make them essential in various industries such as cosmetics, pharmaceuticals, and detergents.^1,2^ In the cosmetic and detergent industry, surfactants are used in products such as dishwashers, cleaners, shampoos, lotions, soaps, body gels, cleansing wipes, micellar water, and creams to facilitate the mixing of ingredients and improve application and absorption on the skin and hair.^3^ All of these formulations incorporate one or more surfactants that provide both high performance and phase stability.

Despite their indisputable usefulness, some surfactants can cause skin irritation, a significant problem especially in daily use products that may be exposed to the skin, such as cosmetics, detergents, and personal care products. The irritation is due to the surfactants’ ability to disrupt the skin’s natural lipid barrier, which can lead to dryness, inflammation, and contact dermatitis. In addition to their potential to cause skin irritation, surfactants may also pose environmental risks. Their limited biodegradability can lead to the contamination of groundwater and negatively impact aquatic ecosystems, including flora and fauna, through the accumulation of persistent compounds.^4^

The prediction and reduction of dermal irritation caused by surfactants are crucial for developing safe and effective surfactant-based formulated products. This goal can be achieved by implementing predictive models based on computational techniques, which allow for the evaluation of the potential irritability of new compounds before their commercial use.^5^ Predicting skin irritation not only enhances consumer safety but also reduces development costs by minimizing the need for advanced-stage testing. Identifying key chemical properties that influence potential irritability allows formulators to design surfactants with improved safety profiles, aligning with the growing demand for eco-friendly and skin-friendly products.

The field of computational chemistry has become increasingly predictive in the twenty-first century, with applications spanning from catalyst development for greenhouse gas conversion and materials discovery for energy harvesting and storage to computer-assisted drug design. The modern chemical simulation toolkit enables scientists to anticipate the properties of a compound with reasonable accuracy before it is synthesized in the laboratory. In recent years, high-throughput computational screening has become a routine practice, allowing researchers to calculate the properties of thousands of compounds in a single study.^6^

Statistical classification methods are essential in the field of chemistry for modeling laboratory results. These methods allow researchers to categorize and interpret complex experimental data, leading to more accurate predictions and insights. By applying statistical classification, chemists can identify patterns and relationships within the data, optimize experimental conditions, and improve the efficiency of chemical processes. This approach enhances the ability to predict the properties of new compounds and materials, making it a valuable tool for innovation and development in chemical research. Decision trees are predictive models that use a tree structure to transform observations about an item (represented in the branches) into conclusions about the value of an item (represented in the leaves). In chemistry, decision trees are applied for the determination of reaction mechanisms, the identification of the most probable steps in a complex chemical reaction, and the evaluation of the mechanical and electrical properties of new materials based on their structure and composition.^7^

To develop effective predictive models in toxicology, enough in vivo and in vitro assays are crucial. Despite the abundance of chemical, biological, and toxicological data available, only a small portion can be directly utilized for quantitative toxicity prediction.^8^ The primary data sources encompass three major domains: 1) Chemical data: this includes information on chemicals, such as chemical descriptors, physical and chemical properties, and molecular structures, which are fundamental for building predictive models. 2) In vivo toxicology data: these data are derived from experiments or studies conducted on live organisms and are scattered across various sources like scientific articles, internal company reports, governmental documents, and institutional services. Integrating this information into publicly accessible data sources through proper extraction, curation, and preprocessing is challenging but extremely valuable. 3) In vitro data: due to its relatively low cost, in vitro cell and molecular biology technology have gained significant attention from toxicology researchers. In vitro research is more suitable for understanding biological mechanisms of action compared with in vivo methods. Combined with chemical information, in vitro data are used to predict in vivo toxicity and prioritize animal testing.

Integrating these data sources allows for the development of more accurate predictive models, enhances the understanding of toxicological mechanisms, and reduces the reliance on animal testing.

In relation to the publicly available toxicology data, there are some databases such as OpenTox, Tox21, OECD Qsar Toolbox, ChEMBL, and ToxCast that mainly include data from ISSCAN, IDEA AMBIT, JRC PRS, EPA DSSTox, ECETOC skin irritation, LLNA skin, and the Bioconcentration Factor (BCF). The additional information for chemical structures can be collected from public sources such as Chemical Identifier Resolver, ChemIDplus, and PubChem.^8^ Despite the availability of public toxicology data, the lack of skin irritation data on surfactants is very important, particularly for surfactant mixtures usually present in cosmetic, detergent, and personal care formulations for which skin irritation data are very limited and partial.

Although the fact that the limited availability of public data on dermal irritation of surfactants may be a limitation when it comes to obtaining sufficient data to be used effectively in computational models, and that the experimental obtaining of this data is very expensive and time-consuming, there are studies that indicate that the use of a set of smaller data sets (experimental or obtained from public databases) can allow the extraction of greater knowledge,^6^ fundamentally due to prior data processing, the elimination of errors, and the interpretability of the results.

Some previous studies in the literature have applied various methods to relate the potential irritation caused by surfactants on skin and eyes to their characteristic parameters, such as structure, nature, toxicity, or CMC.^9–11^ Computational approaches have also been used to develop predictive models for general skin sensitizers such as fragrances, cosmetics, pesticides, or metals.^12^ For example, recent studies have applied machine learning (ML) methods to predict the irritancy of pesticides, chemicals, cosmetics, and ophthalmic drugs using different algorithms, including logistic regression, Naïve Bayes, k-nearest neighbor, support vector machine, random forest, extreme gradient boosting (XGB), and neural networks.^13,14^ In another recent study, chemical irritancy data from public databases were used to develop quantitative structure–activity relationship (QSAR) models based on recurrent neural networks (RNNs) to classify skin irritation caused by chemical compounds.^15^ Huang et al.^16^ proposed predictive models built with five ML algorithms combined with six molecular representations. Their best-performing models achieved an accuracy of up to 97.1% using more than 3,170 bibliographic records on chemical irritancy. Despite these recent advances, a comprehensive and focused study on the development of predictive models specifically based on the parameters and particular characteristics of surfactants, also including surfactant mixtures commonly found in commercial cosmetic products, has not yet been undertaken.

Therefore, the main objective of this work is the development of predictive models for the skin irritation of anionic and nonionic surfactants using a set of experimental data and computational techniques. The study aims to apply and compare the efficacy of statistical classification methods on a data set generated experimentally in the laboratory using the in vitro zein method. Additionally, it seeks to identify the key chemical properties that influence dermal irritability. The generated models enable the classification and prediction of the irritability of surfactants and their mixtures.

This study presents two novel aspects: 1) In vitro skin irritation assessment of individual surfactants and mixtures and production of a skin irritation data set and 2) Development of classification and regression models of skin irritation based on statistical classification methods (trees, multiple linear regression [MLR]) that were applied to develop skin irritation models including properties of the surfactants and mixtures such as HLB and structural characteristic parameters.

This study not only provides an innovative methodology for predicting skin irritation from surfactants but also establishes a framework for future research in improving product safety and efficiency in various industries. By applying advanced computational techniques, the results are expected to provide a deeper understanding of surfactants and contribute to the development of safer and more sustainable products. A flowchart is presented below showing the main methodological stages of the study, from the selection of surfactants to the development and evaluation of the predictive models (Figure 1).

**Figure 1:**
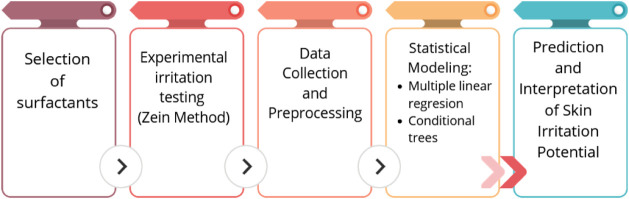
Flowchart of the methodological stages.

## 2 Materials and Methods

### 2.1 Materials

In this work, a total of 20 commercial surfactants have been studied: three ether carboxylic acid derivatives with different alkyl chains and degrees of ethoxylation (Kao Chemicals Europe, S.L.U., Germany), three amine-oxide-based surfactants with structural differences in their hydrophobic alkyl chains (Kao Chemicals Europe, S.L.U., Germany), three alkylpolyglucosides with different alkyl chains and average numbers of glucose units per alkyl radical provided by Sigma-Aldrich (USA), two fatty-alcohol ethoxylates with different alkyl chains and degrees of ethoxylation (Kao Chemicals Europe, S.L.U., Germany), four polyoxyethylene glycerol esters with different degrees of ethoxylation (Kao Chemicals Europe, S.L.U., Germany), Sodium Lauryl Sulfate (Kao Chemicals Europe, S.L.U., Germany), an alcohol ethoxylate (Kao Chemicals Europe, S.L.U., Germany), zinc coceth sulfate (Zschimmer and Schwarz, Germany), zinc dodecyl hydrogen disulfate (Zschimmer and Schwarz, Germany), disodium capryloyl glutamate (Zschimmer and Schwarz, Germany); and disodium lauroyl glutamate (Zschimmer and Schwarz, Germany). The characteristics of the surfactants tested, such as chemical name, surfactant family, ionic character, alkyl chain length, degree of ethoxylation, and HLB, are listed in Table 1. According to the supplier’s specifications and depending on the molecular structure, these surfactants exhibit a wide range of properties suitable for industrial applications.

**Table 1:** Characteristic Parameters of the Surfactants Tested^a^

Chemical name	Family	Character	*R*	*E* or DP	HLB
*Laureth-4 Carboxylic acid*	Polyethoxylated alkyl ether carboxylic acid	Anionic	12–14	*E*: 3	17.09
*Laureth-11 Carboxylic acid*	Polyethoxylated alkyl ether carboxylic acid	Anionic	12–14	*E*: 10	20.35
*Capryleth-6 Carboxylic acid*	Polyethoxylated alkyl ether carboxylic acid	Anionic	8	*E*: 5	16.33
*Myristamine Oxide*	oxide-based surfactants	Nonionic	14	-	23.40
*Lauramine Oxide*	oxide-based surfactants	Nonionic	12	-	24.35
*Cocamidopropylamine Oxide*	oxide-based surfactants	Nonionic	12	-	25.77
*Capryl Glucoside*	Alkylpolyglucosides	Nonionic	9.3	DP: 1.42	13
*Lauryl Glucoside*	Alkylpolyglucosides	Nonionic	11.7	DP: 1.59	11.2
*Coco Glucoside*	Alkylpolyglucosides	Nonionic	11	DP: 1.35	11.9
*Polyoxyethylene(6) Alkyl(C*_*8–12*_ *) Ethers*	Fatty alcohol ethoxylates	Nonionic	10	*E*: 5.2	13.13
*Polyoxyethylene(11) Alkyl(C*_*12–14*_ *) Ethers*	Fatty alcohol ethoxylates	Nonionic	12.6	*E*: 9.9	14.71
*Glycereth-2 Cocoate*	Polyoxyethylene glycerol ester	Nonionic	12	*E*: 2	11.3
*Glycereth-6 Cocoate*	Polyoxyethylene glycerol ester	Nonionic	12	*E*: 6	12.5
*Glycereth-7 Cocoate*	Polyoxyethylene glycerol ester	Nonionic	12	*E*: 7	14.7
*Glycereth-17 Cocoate*	Polyoxyethylene glycerol ester	Nonionic	12	*E*: 17	15
*Sodium lauryl sulfate*	Alkyl sulfate	Anionic	12–14	-	40
*Zinc Coceth Zulfate*	Ethoxylated sulfate	Anionic	12–14	*E*: 3	-
*Zinc Dodecyl Hydrogen Disulfate*	Alkyl sulfates	Anionic	12	-	-
*Disodium Capryloyl Glutamate*	Glutamate	Anionic	8	-	-
*Disodium Lauroyl Glutamate*	Glutamate	Anionic	12	-	-

a *R*: Alkyl chain length; *E*: degree of ethoxylation; DP: average number of glucose units per alkyl radical; HLB: Hydrophilic–Lipophilic Balance.

Ether carboxylic acid derivative surfactants improve the foaming quality of the detergent, reducing the irritation level, and therefore they are used as cosurfactants in detergents and surfactant-based formulations that must be in contact with the skin.^17^ Amine-oxide-based surfactants possess several valuable properties, such as enhancing and stabilizing foam in blends with other amphoteric or anionic surfactants and thickening due to their strong dipolar moment, which structures the surfactant phase. They also increase resistance to oxidation and improve compatibility with the skin and hair. These characteristics make them suitable for use in a variety of industrial, household, and cosmetic products, including detergents, dishwashing liquids, antistatic preparations, shampoos, hair conditioners, and shaving foams. In the case of alkylpolyglucosides, their most notable property is their excellent skin compatibility under typical use conditions, which has led to their widespread use as the primary or cosurfactant in various consumer product formulations.^18^ This compatibility makes them ideal for applications in laundry and dishwashing detergents, cleaning products, cosmetic preparations, and food technology.^19^ Fatty alcohol ethoxylates are the most economically significant group of nonionic surfactants, extensively used in both domestic and commercial detergents, household cleaners, and personal care products. Their versatility, high performance, and cost-effectiveness make them a popular choice in various cosmetic and personal care products, including skin care items, shampoos, and body creams.^20^ Polyoxyethylene glycerol esters are nonionic surfactants derived from vegetable sources, such as cocoa oil, that are commonly used in laundry detergents and household products. They offer several advantages, including low foaming power, high active content, good stability, and high biodegradability,^21^ low toxicity^22^ and low potential for eye and skin irritation. Their compatibility with other surfactants and enzymes and their properties make them ideal for use in laundry pods, concentrated detergents, hand dishwashers, cosmetics, and personal care products. Additionally, they can function as emulsifiers or fragrance solubilizers.^23^

Sodium lauryl sulfate is an anionic surfactant naturally sourced from coconut and/or palm kernel oil. It primarily consists of a blend of sodium alkyl sulfates with lauryl being the dominant component. It is a surfactant used in personal care products due to its foaming, cleansing, emulsifying, moisturizing, and antibacterial properties. Its ability to generate foam makes it ideal for shampoos, shower gels, and toothpastes, as it is associated with the feeling of cleanliness, while its cleansing action facilitates the removal of oils and residues. However, its ability to strip the skin of its natural oils gives it a high potential for skin irritation, especially in sensitive skin or at high concentrations.^24,25^ Additionally, as an emulsifying agent, it helps maintain the stability of products, such as creams and lotions. Its antibacterial activity helps prevent skin infections and promotes oral hygiene, which makes it useful in acne treatments.

Zinc coceth sulfate and zinc dodecyl hydrogen disulfate possess strong antimicrobial properties, which can help reduce bacterial growth on the skin, making them excellent ingredients in acne treatment products and other skincare formulations aimed at reducing skin irritation.^26^ Their anti-inflammatory properties help to soothe irritated skin and reduce redness and swelling. These surfactants can aid in soothing irritated skin and promoting faster healing of minor wounds and inflammation, making them excellent ingredients in skincare products such as lotions and creams. Disodium capryloyl glutamate and disodium lauroyl glutamate are known for their gentle cleansing properties, making them ideal for formulations designed for sensitive skin and baby care products. Their mildness helps reduce the risk of irritation and dryness.^27^

The typical surfactant system found in commercial formulations consists of mixtures of nonionic and anionic surfactants. Mixtures of surfactants can exert synergistic effects in the final formulation, improving performance, modulating foaming, and reducing the potential for skin irritation.^28–31^ In practice, surfactant mixtures are often preferred over single surfactants; consequently, binary mixtures of these surfactants in a 1:1 weight/weight ratio have been incorporated into this study.

### 2.2 Methods

#### 2.2.1 Irritation Potential by the Zein Method

The irritant potential of the surfactants and their mixtures was determined by a modified version of the zein method developed by Bailón et al.^30^ and Lechuga et al.^29^ The method, which consists of three stages, determines the amount of solubilized protein (zein) in a surfactant-containing solution: 1) denaturation of zein, 2) oxidation and mineralization of organic matter, and 3) nitrate content measurement. All experiments were performed at room temperature. Data are the averages of at least three repeats.

For these measurements, the final concentration of each component (whether pure or in a mixture) is set at 0.5%, 1%, and 2% by weight. The formulation is ultimately tested at a 1% active surfactant content, which reflects a typical dosage used in cosmetic and personal hygiene products that come into direct contact with the skin. This concentration is therefore commonly employed for comparing dermal irritation outcomes.

The zein number (ZN), mg of nitrogen released per 100 g of surfactant solution, is calculated as

1 ZN=c/10

Where *c* is the concentration of nitrogen measured spectrophotometrically in parts per million (mg/1000 mL). Considering that the density of the testing solution is 1 g/mL, *c* would be finally expressed as mg/1000 g. Two control cases have been implemented for validation, a negative control using deionized water and a positive control using sodium lauryl sulfate (SLS) at 1% wt.; SLS is a well-known standard surfactant with high irritant potential.

#### 2.2.2 Statistical Modeling

In this study, in addition to multiple regression, conditional trees were performed to analyze the data. Conditional trees, a variation of regression trees, split the data into homogeneous groups based on predictor variables to predict a response variable^31,32^ and use statistical tests to ensure splits are significant, enhancing interpretability.^33^ These methods were applied to model the relationship between surfactant characteristics and their irritability values (ZN number), allowing for accurate predictions and identification of key influencing factors.^34^ The analysis was conducted using the R packages (R Core Team. (2021)). R: A language and environment for statistical computing. R Foundation for Statistical Computing. < https://www.R-project.org/> “MASS” and “party”.^33,35^

Statistical analyses were conducted to explore the relationships between the response variable ZN number and the characteristic parameter HLB; character of the surfactant with the levels: A (Anionic), AA (Anionic–Anionic mixture), ANI (Anionic and Nonionic mixture), NI (Nonionic), and NINI (Nonionic and Nonionic mixture); and concentration with levels: 0.5%, 1%, and 2% wt. Descriptive statistics were calculated for the ZN number and HLB, summarizing the mean, standard deviation, range, and sample sizes for each combination of the factors. A generalized linear model (GLM) with a Gamma distribution and an identity link function was applied to the ZN number, as the distribution was clearly non-Gaussian and better captured by the Gamma family. The predictors included HLB, character, and concentration. Conditional inference trees were also constructed to identify interactions and thresholds in the relationships between the predictors and the ZN number.

## 3 Results and Discussion

### 3.1 Skin Irritation Test

The Zein number provides an indication of potential surfactant-induced skin irritation. According to the scale established by Götte (1964),^36^ surfactants can be classified as nonirritant, moderate irritant, irritant, or strong irritant. The final concentration of each component, whether individual or in a mixture, was set at 0.5%, 1%, and 2% wt. 92.8% of the surfactants and mixtures tested at the 1% concentration are classified as nonirritating, indicating minimal irritation potential. Anionic surfactants show a higher potential for skin irritation compared to nonionic surfactants, primarily due to their greater ability to solubilize zein protein.^37^ ZN values support this observation, establishing a decreasing order of irritation potential for the tested character of the surfactants and mixtures of them as follows: NI < ANI < NINI < AA < A. The addition of nonionic surfactants to mixtures containing anionic surfactants significantly moderates skin irritation, as evidenced by the reduction in ZN values. The incorporation of mild surfactants significantly enhances dermatological properties. Nonionic surfactants, such as fatty alcohol ethoxylates, effectively reduce the irritant effects of anionic surfactants like polyethoxylated alkyl ether carboxylic acid. This mitigation is primarily attributed to their ability to lower charge density and minimize protein solubilization, both of which are critical factors linked to irritation. The concentration of surfactants has a notable influence on skin irritation values. Across all surfactants and their mixtures, ZN values consistently increased at the 1% and 2% concentration compared to 0.5%, highlighting a dose-dependent response in irritation potential. The increase in ZN from 0.5% to 1% is more pronounced in the individual anionic surfactants polyethoxylated alkyl ether carboxylic acids, reinforcing the idea that anionic surfactants have a higher irritation potential, while combinations with a fatty alcohol ethoxylate appear to moderate this effect.

Over the years, various theories have been proposed to explain how surfactants penetrate human skin, depending on their chemical properties.^38^ It is widely accepted that when surfactant monomers contact the skin surface, they interact with the keratin protein in the stratum corneum (SC), causing denaturation of its α-helical structure. This denaturation exposes sites where water molecules can bind, leading to swelling of the SC. As a result, the degraded protein structure facilitates the easy removal of proteins from the skin and their solubilization in the solution, allowing amphiphilic compounds, other chemicals, and pathogens to penetrate deeper layers of the SC.^39^ While the interaction of surfactants with skin proteins is a significant factor in skin irritation, it is only one of several mechanisms proposed in the literature. Surfactants can also interact with intercellular lipids, sebum, and living skin cells.

The critical micelle concentration (CMC) is a fundamental physicochemical parameter that influences surfactant behavior in solution. Surfactants with lower CMC values tend to form micelles at lower concentrations, which can reduce the number of free monomers available to interact with skin proteins, potentially decreasing irritation. However, this relationship is complex and may be modulated by additional factors, such as molecular structure, ionic character, and formulation environment. Therefore, while CMC provides useful insight into surfactant aggregation behavior, its role in predicting irritability should be considered alongside other structural and functional parameters.

### 3.2 Descriptive Statistics

This study provides a descriptive statistical analysis of ZN values obtained via the zein method, exploring the impact of the HLB on the solubilization properties of individual surfactants and their mixtures. The HLB is a critical parameter that significantly influences the ability of ionic surfactants to interact with globular proteins like zein. Surfactants with optimal HLB values enhance the balance between their hydrophilic and lipophilic properties, maximizing their solubilization capacity. Ionic surfactants with the appropriate HLB interact better with oppositely charged globular proteins, which is crucial for protein denaturation and improving their solubility.^40^

The HLB of the surfactant mixture was determined using the geometric mean of the individual HLB values rather than the conventional arithmetic mean. This method yielded a closer correlation with the experimental data, offering a more accurate representation of the mixture’s behavior. Additionally, the geometric mean is particularly suitable for systems where there is a multiplicative effect, as occurs in the interaction of surfactants, where the relative contribution of each component is not simply additive but proportionally influences the final properties of the mixture. The equation for calculating the geometric mean is

2 $$HLB_{mixture}=\sqrt{HLB_{1}\cdot HLB_{2}}$$

where HLB_1_ and HLB_2_ represent the HLB values of the individual surfactants in the mixture.

At low concentrations, surfactant molecules bind to the protein in a specific and noncooperative manner, gradually increasing the number of surfactant molecules bound per protein molecule. As the concentration of surfactants increases, a phase of cooperative and nonspecific binding is observed, characterized by a rapid increase in the number of bound molecules, associated with protein unfolding.^41^

Increasing the surfactant concentration improves its binding effectiveness, resulting in better solubilization and higher mean values of ZN. Higher concentrations, especially at 2%, are associated with increased mean values of ZN, with the highest observed values corresponding to the AA character level. This phenomenon is due to the anionic nature of the mixture and the well-documented ability of ionic surfactants to strongly interact with oppositely charged globular proteins, causing their denaturation.^42^ However, as the concentration of surfactants continues to rise, a saturation phase is eventually reached, where no further binding of the surfactant to the protein occurs, leading to the formation of micelles in the solution. Nevertheless, in the region prior to saturation, the solubilization of zein is significantly enhanced, explaining the higher observed ZN values. This behavior is particularly relevant in formulations designed to maximize cleaning effectiveness and the stability of the mixture, as greater solubilization of the proteins can improve the detergent capacity of the system.^37^

Table 2 summarizes the mean values of ZN and HLB across different surfactant ionic characteristics and concentrations. As shown, ZN values increase with concentration for all surfactant types, with the highest irritation potential observed in anionic (A) and anionic–anionic (AA) mixtures at 2% wt. In contrast, nonionic (NI) and mixed nonionic/nonionic (NINI) surfactants exhibit lower ZN, suggesting reduced protein solubilization and milder irritation profiles. The HLB values also vary by surfactant character, with anionic surfactants presenting broader ranges, which may contribute to their stronger interaction with zein protein. These trends reinforce the importance of both concentration and ionic nature in predicting skin irritation potential. Although less explicitly, the skin irritation potential is also related to the different functional groups that make up the polar portion of the surfactant molecules, which play a key role in determining both the HLB of the molecule and its ZN. For instance, nonionic surfactants with a regular alkyl chain incorporating an amine oxide in the polar head exhibit high HLB values, which correspond to higher ZN values. In contrast, alkyl polyglucosides, in which glucose units serve as functional groups, display lower HLB values and relatively modest irritation potential (ZN).

**Table 2:** Mean Values of ZN by Ionic Character and the Concentration and Mean Values of HLB by Ionic Character

		ZN			HLB		
Character	Conc.	Mean (sd)	Min-Max	*N* (^a^)	Mean (sd)	Min-Max	*N* (^a^)
A	0.5%	154.2 (41.7)	110.7–248.0	8 (0)	21.1 (7.8)	16.3–40.0	24 (0)
	1%	330.4 (125.5)	183.5–531.0	8 (0)			
	2%	496.3 (106.8)	383.6–637.0	5 (3)			
AA	0.5%	126.8 (13.6)	117.2–136.4	2 (0)	17.7 (1.4)	16.7–18.7	6 (0)
	1%	403.3 (116.8)	320.7–485.9	2 (0)			
	2%	483.6 (47.0)	450.4–516.8	2 (0)			
ANI	0.5%	65.3 (61.6)	0.5–189.0	29 (6)	17.8 (4.0)	12.5–32.1	102 (1)
	1%	99.0 (88.2)	3.1–366.5	35 (0)			
	2%	239.6 (149.1)	43.1–512.8	15 (20)			
NI	0.5%	90.2 (120.0)	2.1–325.1	18 (2)	16.5 (5.5)	11.2–25.8	60 (0)
	1%	142.2 (178.5)	12.8–564.0	20 (0)			
	2%	192.3 (214.1)	34.0–624.0	10 (10)			
NINI	0.5%	75.1 (60.7)	21.3–198.3	17 (6)	18.9 (2.5)	16.3–25.1	69 (0)
	1%	135.9 (72.3)	9.8–255.1	19 (4)			
	2%	447.0 (75.3)	367.5–517.2	3 (20)			

a denotes the number of missing values; sd (standard deviation).

ZN values increase with concentration; however, there are missing values, particularly at a concentration of 2%, which resulted in fitting the proposed models with 192 cases out of the original 351. These missing cases could be imputed later by using the fitted models, allowing for a second phase where the accuracy of the model estimation can be assessed.

A multiple linear regression analysis was conducted to evaluate the influence of surfactant ionic character, concentration, and HLB on the skin irritation potential expressed as ZN. The model revealed several statistically significant predictors (*p* < 0.05), indicating that these physicochemical parameters contribute meaningfully to dermal irritation outcomes (Table 3). Among the surfactant types, anionic surfactants showed a significant negative effect (*B* = −100.062, *p* = 0.005), suggesting a higher potential for skin irritation compared to the reference group. Similarly, combinations of anionic/nonionic (*B* = −237.978, *p* < 0.001), nonionic/no-ionic (*B* = −211.914, *p* < 0.001), and nonionic (*B* = −172.153, *p* < 0.001) surfactants were also significant, each associated with a reduction in the dependent variable, potentially reflecting differential irritation profiles. In contrast, the combination of anionic/anionic surfactants did not yield a significant effect (*p* = 0.833), indicating a negligible contribution to the variance in irritation under the tested conditions.

**Table 3:** Multiple Linear Regression Analysis of Surfactant Character, Concentration, and HLB on ZN

	Unstandardized regression coefficient (*B*)	Standard error	Standardized regression coefficient (β)	*t*	*p*
A	–100.062	35.152	-	–2.847	0.005
AA	–8.778	41.467	-	–0.212	0.833
ANI	–237.978	31.239	-	–7.618	<0.001
NI	–172.153	28.142	-	–6.117	<0.001
NINI	–211.914	33.783	-	–6.273	<0.001
Conc. 1%	67.830	15.262	-	4.443	<0.001
Conc. 2%	173.868	20.236	-	8.592	<0.001
HLB	14.68	1.427	0.503	10.287	<0.001

Surfactant concentration was positively associated with irritation: both 0.01% (*B* = 67.830, *p* < 0.001) and 0.02% (*B* = 173.868, *p* < 0.001) concentrations were significant predictors, with the higher concentration exerting a stronger influence. Furthermore, mean HLB values were also significantly associated with increased irritation potential (*B* = 14.680, β = 0.503, *p* < 0.001), underscoring the role of surfactant polarity in dermal response.

Collectively, these results emphasize that both the ionic nature and formulation parameters of surfactants, particularly HLB and concentration, are critical determinants of skin irritation potential and should be carefully considered in the design of dermally applied products.

### 3.3 Model Analysis

#### 3.3.1 Generalized Linear Model

One of the models considered was a multiple regression model. This model relates surfactant characteristics (HLB), their concentrations (0.5%, 1%, and 2%), and the type of surfactant (anionic and nonionic) and their mixtures (anionic/anionic, anionic/nonionic, nonionic/nonionic) to the dependent variable (ZN). For mixtures, the HLB value is the geometric mean of the HLB for the individual surfactant. The model explains 80% of the variability (adjusted *R* ^2^ = 0.801), with all factors being significant except for the anionic/anionic mix regarding the average ZN. The coefficients indicate that all considered factors increase the ZN as concentrations rise, as expected, and that some mixtures are more effective in reducing the ZN.

To provide a better fit for the data and considering the non-Gaussian nature of the response variable ZN, a Generalized Linear Model (GLM) was fitted using a Gamma distribution with an identity link function (dispersion parameter of 0.524; null deviance: 249.91, 191 d.f.). The Gamma distribution was evaluated and found suitable for modeling the response (Figure 2) (Anderson–Darling test, *p* = 0.8051).

**Figure 2:**
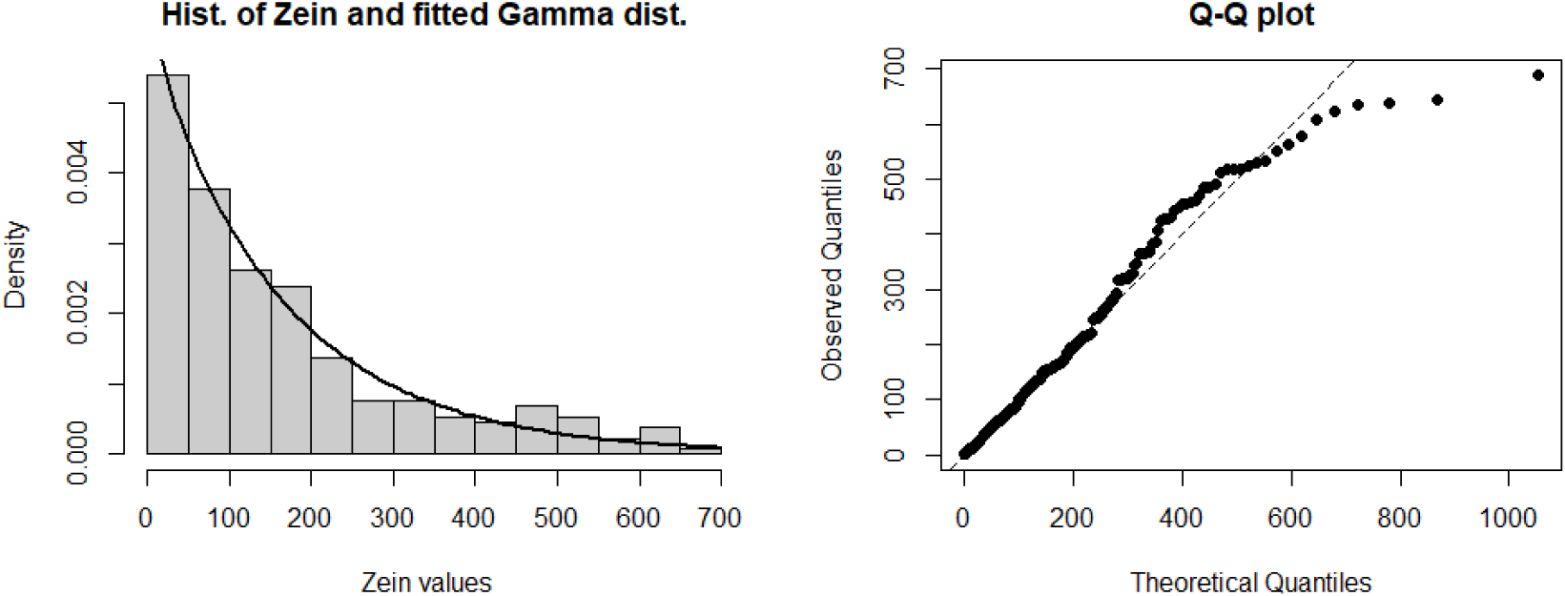
ZN with fitted Gamma model and Q–Q plot for the goodness-of-fit.

Figure 3 demonstrates a strong fit for the data under consideration.

**Figure 3:**
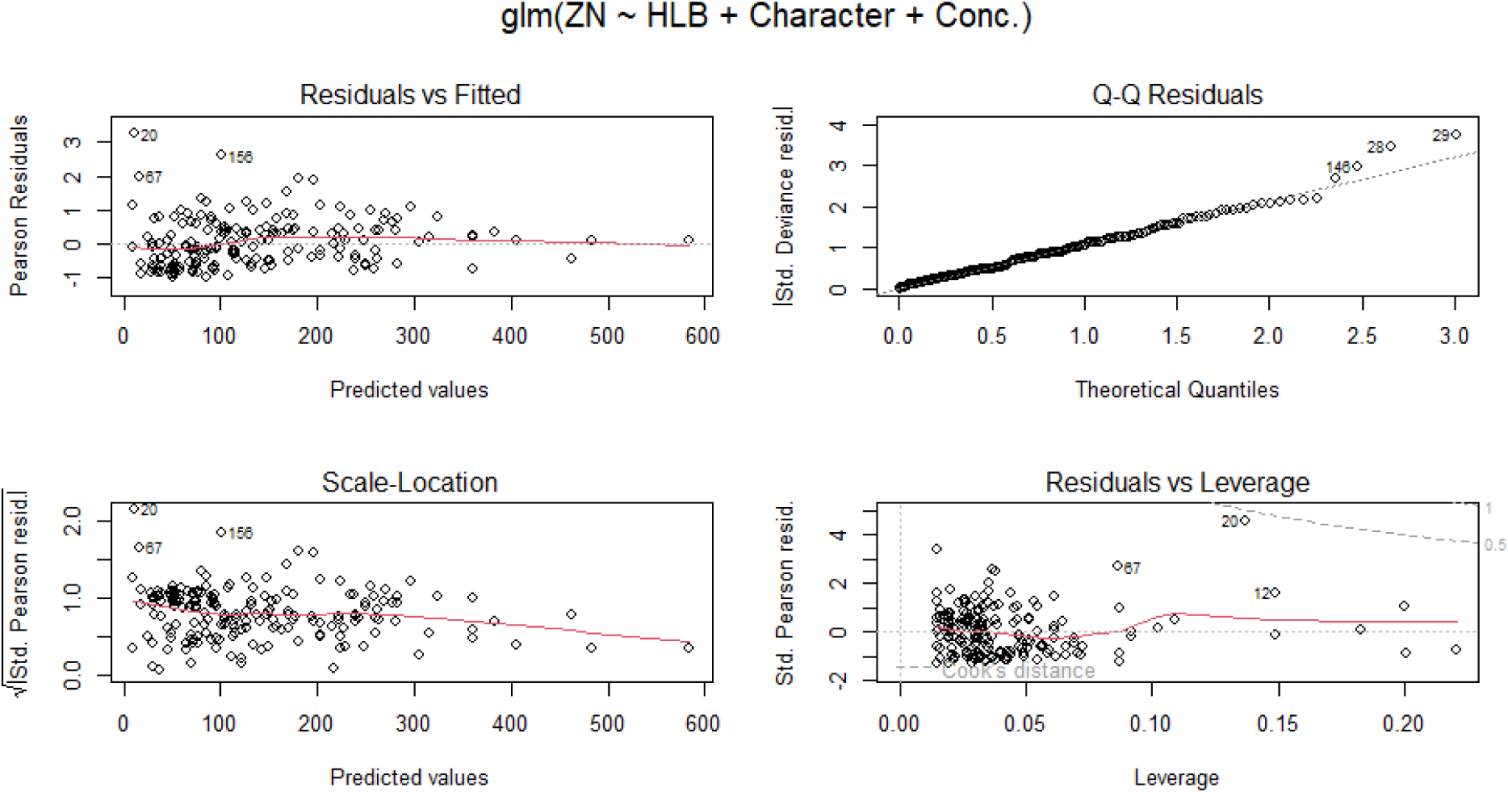
Diagnostic plots for the generalized linear model of ZN with HLB, character, and concentration.

The estimated coefficients for the GLM models are listed in Table 4.

**Table 4:** Coefficients for the GLM Models

Variable	Estimate (β)	Std. Error	*t* value	*p*
(Intercept)	28.554	46.588	0.613	0.5406
HLB	10.222	1.446	7.070	<0.001
Character: AA	63.558	98.869	0.643	0.5211
Character: ANI	–133.491	39.332	–3.394	<0.001
Character: NI	–128.807	39.874	–3.230	0.0014
Character: NINI	–136.607	40.146	–3.403	<0.001
Conc.: 1%	20.396	6.961	2.930	0.0038
Conc.: 2%	119.820	27.502	4.357	<0.001

The fitted model indicates that HLB has a significant positive effect on ZN (β = 10.222, *p* < 0.001), highlighting a strong direct association. The character of the surfactant has significant effects, with ANI (β = −133.491, *p* < 0.001), NI (β = −128.807, *p* = 0.001), and NINI (β = −136.607, *p* < 0.001) associated with notable reductions in ZN compared to the baseline level A. The concentration of the surfactant also emerged as a significant factor, with a concentration of 1% (β = 20.396, *p* = 0.004) and a concentration of 2% (β = 119.820, *p* < 0.001) both associated with significant increases in ZN, regarding the base level of 0.5% the effect, being particularly pronounced at a concentration of 2% (Figure 4).

**Figure 4:**
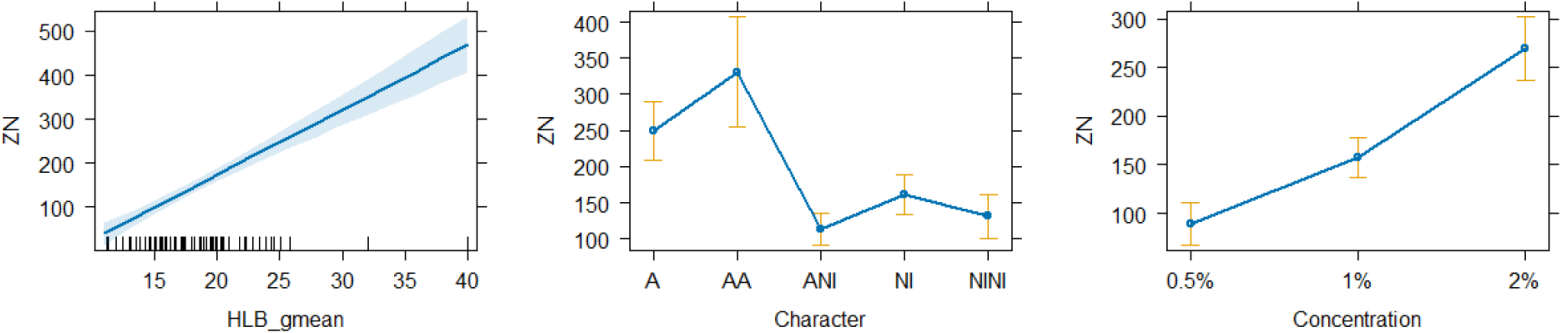
Predictor effect plots for ZN: influence of character, concentration, and compound HLB.

In Figure 4 it can be observed that the prediction interval for the AA mixture, in coherence with Table 3, is excessively wide, primarily due to the reduced number of observations. Concentration, on the other hand, exhibits a behavior that is slightly more than linear. Specifically, when comparing the 2% concentration to 1%, the increase shows a steeper slope compared with the transition from 0.5% to 1%. Additionally, at higher concentrations, variability becomes more pronounced. This suggests that for formulation purposes such concentrations might be avoided in final formulations, as they result in more dispersed predictions. Regarding the HLB, it is also evident that as its value increases, the predicted ZN values rise, though this is accompanied by greater variability in the predictions.

#### 3.3.2 Classification Trees

A conditional tree has been developed to better illustrate the relationships between HLB and the factors considered, highlighting patterns where low ZN values occur. The resulting tree (Figure 5) identified the HLB as the most influential predictor (*p* < 0.001). Specifically, surfactants with HLB values ≤16.261 followed a distinct path in which concentration became the next key determinant: higher concentrations (1% and 2%) resulted in significantly higher ZN (Node 3), whereas lower concentrations (0.5%) were associated with reduced protein denaturation (Node 4).

**Figure 5:**
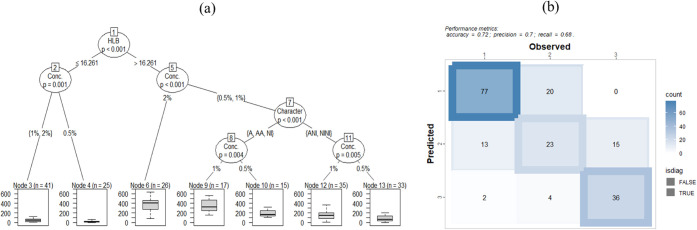
Decision tree model (a) and confusion matrix (b) predicting ZN based on surfactant HLB, concentration, and ionic character.

In contrast, surfactants with HLB > 16.261 followed a more branched path. At this level, concentration again played a critical role (*p* < 0.001), with 2% solutions (Node 6) exhibiting markedly higher ZN. For lower concentrations (0.5% and 1%), the model further split based on surfactant ionic character (*p* < 0.001). Anionic (A), anionic–anionic (AA), and nonionic (NI) surfactants under these conditions showed concentration-dependent effects (Node 9 vs Node 10), with 1% generally leading to higher ZN. On the other hand, anionic–nonionic (ANI) and nonionic–nonionic (NINI) combinations formed a separate branch, where concentration again significantly influenced outcomes (Node 12 vs Node 13; *p* = 0.005), with 1% yielding greater irritation potential.

Overall, the decision tree model clearly highlights the hierarchical influence of surfactant properties on ZN, a proxy for skin irritation potential. HLB emerged as the primary discriminant, with concentration modulating the extent of protein denaturation within HLB-defined categories and ionic character refining the prediction particularly in high-HLB surfactants at low to moderate concentrations. These findings suggest that the structural HLB of a surfactant should be considered as the first-level criterion when evaluating or selecting ingredients for formulations, provided that key functional properties, such as emulsification, wetting, cleansing, or formulation stability, can still be achieved. Low-HLB surfactants may be preferable when the goal is to minimize irritation, whereas high-HLB surfactants require careful formulation, especially with regard to their ionic nature and concentration. The use of combinations of anionic and nonionic surfactants appears to attenuate the expected irritation potential. This effect may be attributed to a reduced surface charge density, which likely diminishes electrostatic interactions between micelles and the zein protein.^38^ The model also highlights that nonionic/nonionic (NINI) mixtures are associated with lower predicted irritation compared to the average values observed for individual nonionic surfactants. This suggests that mixed micellar structures formed by monomers of different surfactants may exhibit reduced protein interactions, potentially due to altered molecular packing or interfacial behavior.

As expected, higher surfactant concentrations consistently led to elevated ZN across all branches of the tree, underscoring the importance of formulating with the lowest effective concentration necessary to achieve the desired function. This approach not only minimizes irritation potential but also offers benefits in terms of formulation cost, environmental impact, and resource consumption.

The classification performance of the decision tree model was evaluated by using a confusion matrix (Figure 5), which shows a moderate to good ability to discriminate between the three ZN classes. The model achieved an overall accuracy of 72%, with a precision of 0.70 and a recall of 0.68, indicating reliable predictive performance in this multiclass context. Class 1 (lowest irritation) was predicted with the highest accuracy, correctly classifying 77 out of 97 cases, while only 20 and 0 were misclassified into Classes 2 and 3, respectively. Class 3 (highest irritation) also showed good discrimination, with 36 correct predictions and relatively few misclassifications (6 in total). However, Class 2 displayed more overlap, with considerable misclassifications into both Class 1 (13 cases) and Class 3 (15 cases), reflecting a greater degree of variability or overlap in intermediate irritation levels.

These results suggest that the tree-based model is particularly effective at distinguishing between low and high irritation potentials, while the intermediate class remains more challenging to classify, potentially due to overlapping physicochemical profiles. This underlines the importance of refining the input variables or employing complementary modeling approaches for enhanced resolution of borderline cases.

## 4 Conclusions

This study successfully highlights the significant interplay between surfactant properties—HLB values, ionic character, and concentration—and their impact on protein denaturation, as represented by the Zein number (ZN). The development of robust statistical models, including multiple regression and classification trees, has provided valuable insights into how these factors influence the skin irritability potential. The multiple regression model demonstrates the predictive power of HLB, surfactant type, and concentration, explaining 80% of the variability in ZN values. Higher concentrations and elevated HLB values are shown to correlate with increased protein denaturation, although variability also rises with these factors. This suggests the importance of formulating products using lower concentrations and optimizing HLB values to reduce the irritation risk. Furthermore, the fitted generalized linear model (GLM) and classification tree models reinforce the hierarchical importance of HLB, with concentration and ionic character playing secondary yet crucial roles.

The findings underscore that surfactant mixtures, particularly combinations of anionic and nonionic surfactants, exhibit reduced irritation potential, likely due to decreased electrostatic interactions and altered micellar structures. The study also emphasizes the benefits of nonionic/nonionic mixtures, which may contribute to safer formulations by minimizing protein interaction effects.

The decision tree model supports the use of low-HLB surfactants when minimal irritability is desired, while high-HLB surfactants require careful design and testing, especially in terms of their ionic composition and concentration levels. By achieving an overall classification accuracy of 72%, the tree-based model demonstrates its reliability in predicting irritation potential, particularly for distinguishing between low and high irritation outcomes. However, refinement is needed to enhance accuracy for intermediate classes, suggesting the integration of complementary modeling techniques or additional variables for further precision.

These results hold critical implications for industries focused on cosmetics, detergents, and pharmaceutical formulations. The predictive tools developed here serve as a valuable resource for optimizing surfactant selection and formulation design and balancing efficacy, safety, and sustainability.

## Data Availability

Data sets used in this study are available at: https://zenodo.org/records/15800001
